# Oral manifestations of Diabetes Mellitus. A systematic review

**DOI:** 10.4317/medoral.21655

**Published:** 2017-08-16

**Authors:** Elisabet Mauri-Obradors, Albert Estrugo-Devesa, Enric Jané-Salas, Miguel Viñas, José López-López

**Affiliations:** 1 DDS, Department of Dentistry and Stomatology. University of Barcelona. L’Hospitalet, Barcelona, Spain; 2 DDS, MD. PhD, Department of Dentistry and Stomatology. University of Barcelona and IDIBELL. Dental Hospital Barcelona University. Spain; 3 PhD, Department Pathology & Experimental therapeutics, University of Barcelona and IDIBELL. L’Hospitalet, Barcelona, Spain

## Abstract

**Background:**

Diabetes Mellitus has become a global epidemic and presents many complications, usually proportional to the degree and duration of hyperglycemia. The aim of this systematic review was to investigate the different oral manifestations associated with Diabetes Mellitus.

**Material and Methods:**

A MEDLINE search for “Diabetes Mellitus and oral manifestations” was performed. A further search was conducted for “diabetes” and its individual oral manifestation. Inclusion criteria were as follows: human clinical studies with a minimum of 30 patients; studies published in relevant scientific journals between January 1998 and January 2016. Nineteen studies fulfilled the inclusion criteria and were analyzed, assessing the strength of scientific evidence according to recommendations made by the Centre for Evidence-Based Medicine, Oxford (OCEBM), which permits adequate assessment of prevalence studies.

**Results:**

A total 3,712 patients (2,084 diabetics) were included in the studies reviewed. Of the 19 studies analyzed, 4 were longitudinal studies and 15 cross-sectional studies. Periodontal disease, periapical lesions, xerostomia and taste disturbance were more prevalent among diabetic patients. An association between diabetes and caries and mucosal lesions proved positive in 5 out of 10 studies.

**Conclusions:**

Despite multiple oral manifestations associated with DM, awareness of the associations between diabetes, oral health, and general health is inadequate. It is necessary for doctors and dentists to be aware of the various oral manifestations of diabetes in order to make an early diagnosis.

** Key words:**Diabetes Mellitus, oral manifestations, oral pathology.

## Introduction

Diabetes Mellitus (DM) is a metabolic disorder characterized by the presence of chronic hyperglycemia accompanied to greater or lesser extent by alterations to carbohydrate, protein, and lipid metabolisms. DM has become a global epidemic, the complications of which significantly impact on the quality of life and longevity of the sufferers, as well as healthcare costs. The number of people with diabetes has increased from 108 million in 1980 to 422 million in 2014. The overall prevalence of diabetes among adults over 18 years of age has increased from 4.7% in 1980 to 8.5% in 2014 and the World Health Organization (WHO) predicts this will increase to 439 million, almost 10% of adults in 2030 (1).

Patients with diabetes present impaired function of polymorphonuclear leukocytes (leukocyte adhesion, chemotaxis, and phagocytosis), impaired bactericidal activity, altered response to exposure to antigens, and alteration to the function of T lymphocytes (2). Many studies have shown a clear link between chronic inflammation and the development of Type 2 Diabetes Mellitus (DM2) (2,3).

Both Diabetes Mellitus type 1 (DM1) and type 2 diabetes (DM2) present numerous possible long-term complications. Epidemiological studies indicate that the severity of diabetic complications is generally proportional to the degree and duration of hyper-glycemia (4). Among the oral manifestations related to DM described are: dry mouth, tooth decay, periodontal disease and gingivitis, oral candidiasis, burning mouth syndrome (BMS), taste disorders, rhinocerebral zygomycosis (mucormycosis), aspergillosis, oral lichen planus, geographic tongue and fissured tongue, delayed wound healing, and increased incidence of infection, salivary dysfunction, altered taste and other neurosensory disorders, impaired tooth eruption, and benign parotid hypertrophy (5). The objective of this review was to provide a systematic overview of the literature on the various oral manifestations that may occur in diabetic patients.

## Material and Methods

This systematic review was conducted in order to answer the question: what are oral the manifestations of diabetes? The review followed guidelines detailed in Preferred Reporting Items for Systematic Reviews and Meta-analysis (PRISMA) (6).

- Database Selection

Two blinded reviewers (JL and EM) made the study selection according to inclusion/exclusion criteria. Both reviewers agreed with the selection of articles.

- Search strategy

A literature search was performed in the MEDLINE database applying the search terms “Diabetes and Oral manifestations,” selecting transverse and longitudinal human studies published between January 1998 and January 2016. A further search was then performed using the terms “Diabetes Mellitus” combined with different oral manifestations: “Dental Caries”; “Periapical lesions”; “Periodontal disease”; “Salivary dysfunction”; “Oral mucosal pathology”; “Taste alteration”; “Burning mouth syndrome.” Other studies were located using the Cochrane database and other academic search engines available via Google, or obtained from the reference lists of review articles.

- Screening and selection

Studies were selected applying the following inclusion criteria: articles published in English, between January 1998 and January 2016, in scientific journals, original research, studies conducted on a human population, with more than 30 patients. Exclusion criteria were: animal studies, in vitro studies, studies with fewer than 30 patients, not original papers, systematic reviews, meta-analyses.

- Data extraction and statistical analysis

Data were collected by two independent reviewers (JL and EM). A tab-based data collection protocol was designed for the 19 items included in the review. The review was performed following guidelines detailed in the PRISMA statement, aimed at to improving the quality of systematic reviews and meta-analyses (6). The reviewers crosschecked all extracted data. Disagreements were resolved by discussion until consensus was reached.

- Methodological study quality assessment

The quality of the articles was assessed by two independent reviewers. The strength of scientific evidence was rated following recommendations made by the Centre for Evidence-Based Medicine, Oxford (OCEBM), which allows adequate assessment of studies of disease prevalence (7).

## Results

**Figure 1:**
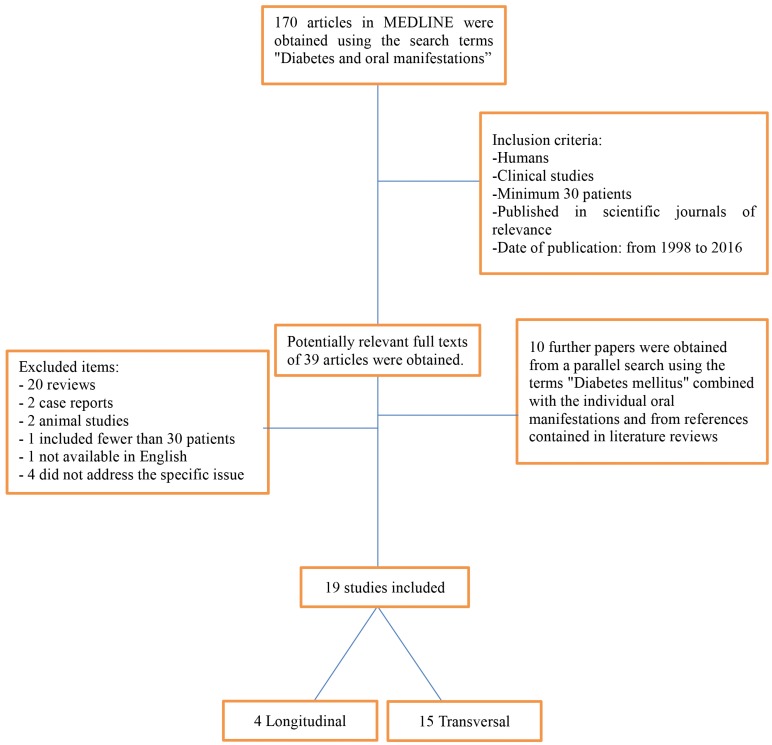
Flow chart diagram.

The search conducted using the MEDLINE database identified 170 published articles. After reading the abstracts, 39 works were selected of which only nine met the inclusion criteria: human studies published between 1998 and 2016, and with a minimum sample of 30 patients with DM1 and/or DM2. Twenty articles were excluded as they were reviews, two because they were case reports, two animal studies, one as it included fewer than 30 patients, one as it was not available in English, and four that did not deal specifically with the oral manifestations of DM. A parallel search applying the search terms “Diabetes Mellitus” in combination with the various relevant oral manifestations, and a search among the references included in literature reviews identified a further ten studies. See Flow chart Diagram in Figure 1.

The final selection of articles included a total of 3,712 patients, of whom 2,084 had diabetes. Of the 19 studies included in the review, four were longitudinal studies and 15 cross-sectional studies. Of these, 14 (74%) found a higher prevalence of oral manifestations in patients with DM (8-21), while the remaining 5 (26%) did not obtain any significant differences between DM groups and control groups of healthy subjects (22-26). Of these, three explored the association of caries with DM (24-26) and two the association of mucosal lesions with DM (22,23).

Two studies dealt with the association between periodontal disease (PD) and DM (8,24), and three the association of periapical lesions with DM (9-11); all these five articles (100%) identified significant differences between patients with DM and control groups of healthy subjects. All studies (100%) investigating xerostomia (18-21), and taste alteration (17) found a higher incidence of these pathologies among diabetic patients compared to non-diabetic patients ( Table 1). As regards other oral manifestations, the results generated some controversy. Of studies examining caries, 40% (12,13) found an association with DM, while 60% did not (24-26); of those that evaluated the presence of mucosal lesions, 50% (14-16) found an association with diabetes and 50% did not (22-24).

**Table 1:**
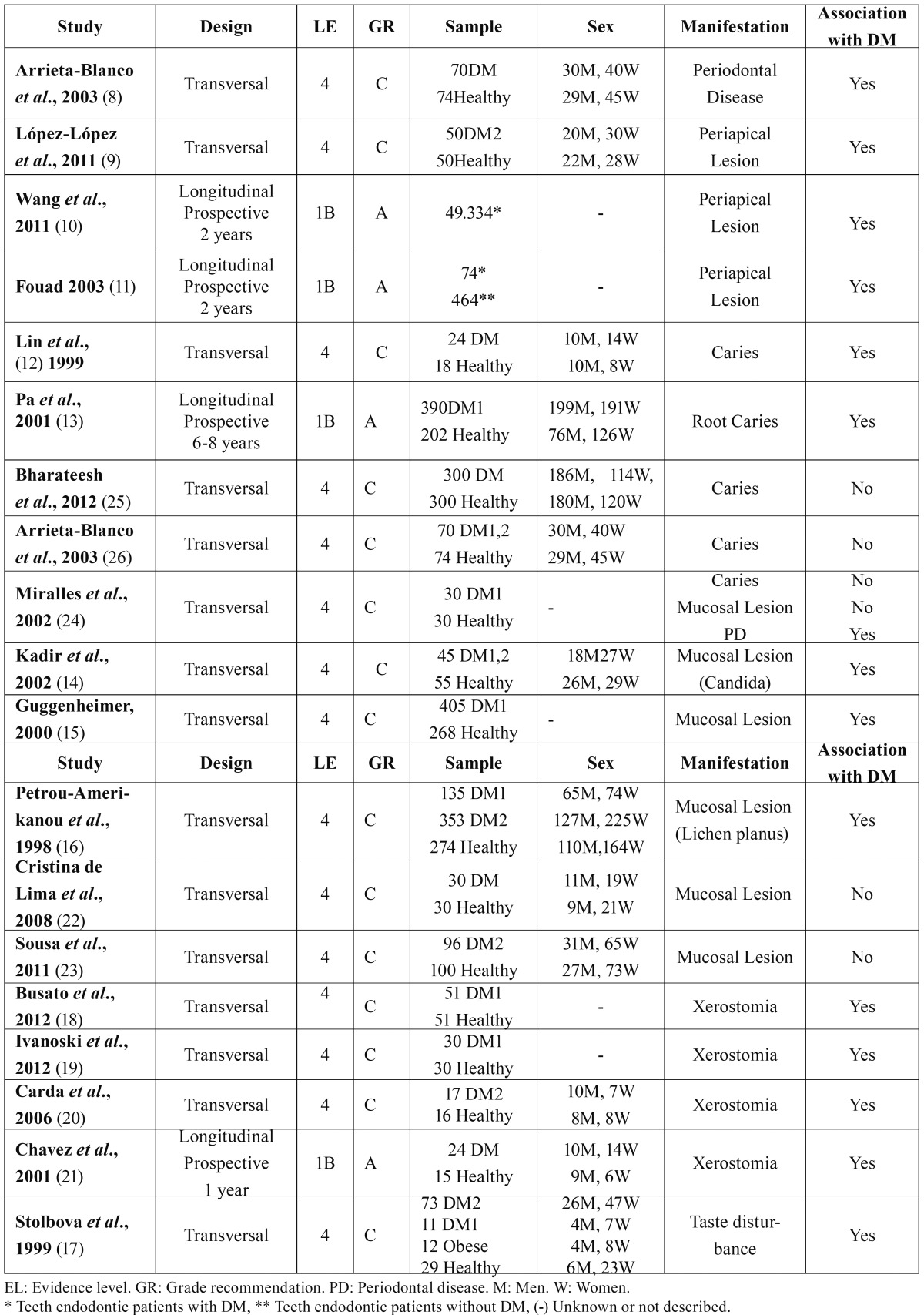
Studies included in the review.

Assessment of the strength of scientific evidence (OCEBM) showed that longitudinal studies presented stronger scientific evidence. Of the 4 longitudinal studies included in this review two investigated the presence of periapical lesions (10,11) (49,334 and 538 teeth treated endodontically, respectively); one the presence of caries (13) (592 patients); and another the presence of xerostomia (21) (39 patients). All these longitudinal studies found the diseases studied to be associated with DM.

## Discussion

- Pathophysiology of oral manifestations

Two mechanisms involved are involved in the pathogenesis of diabetic complications. Firstly, the polyol pathway converts glucose into the enzyme sorbitol byaldose reductase that causes tissue damage and numerous other diabetic complications. Secondly, the formation of advanced glycosylation end products (AGE), whose formation is due to binding of glucose to proteins, lipids and nucleic acids, results in the alteration of structures and functions, in addition to its deposition in specific organs that causes various complications (27). Atheroma deposits are formed in cells, which accumulate in the basal membrane and lumen causing decreased cellular defense capacity and impaired polymorphonuclear leukocyte response (28). This makes diabetic patients more susceptible to infection processes especially when these are caused by anaerobic bacteria due to the reduction of oxygen diffusion through the capillary wall (29).

**Figure 2:**
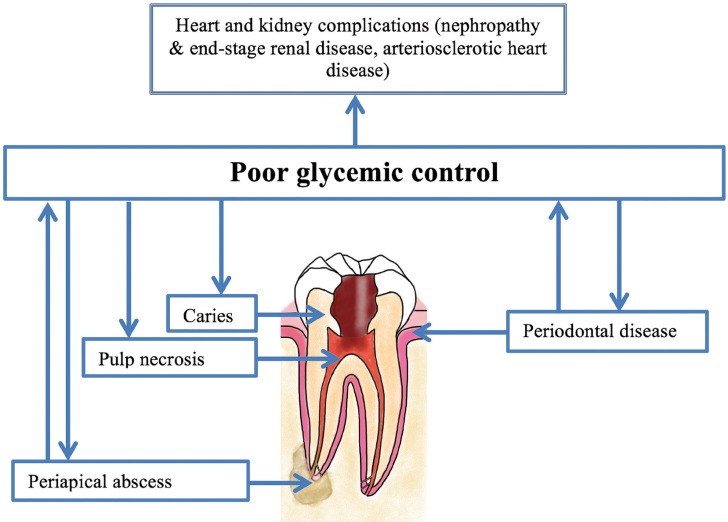
Pathophysiological relationship between diabetes and dental disease. Kudiyirickal adaptation of Kudiyirickal & Pappachan (30).

Figure 2 summarizes the most significant aspects of the pathophysiological relationship between diabetes and dental disease; the figure is based on a diagram proposed by Kudiyirickal MG *et al.* (30).  Table 2 shows the pathophysiology, treatment, and prevention aspects of orofacial diseases related to diabetes (30).

**Table 2:**
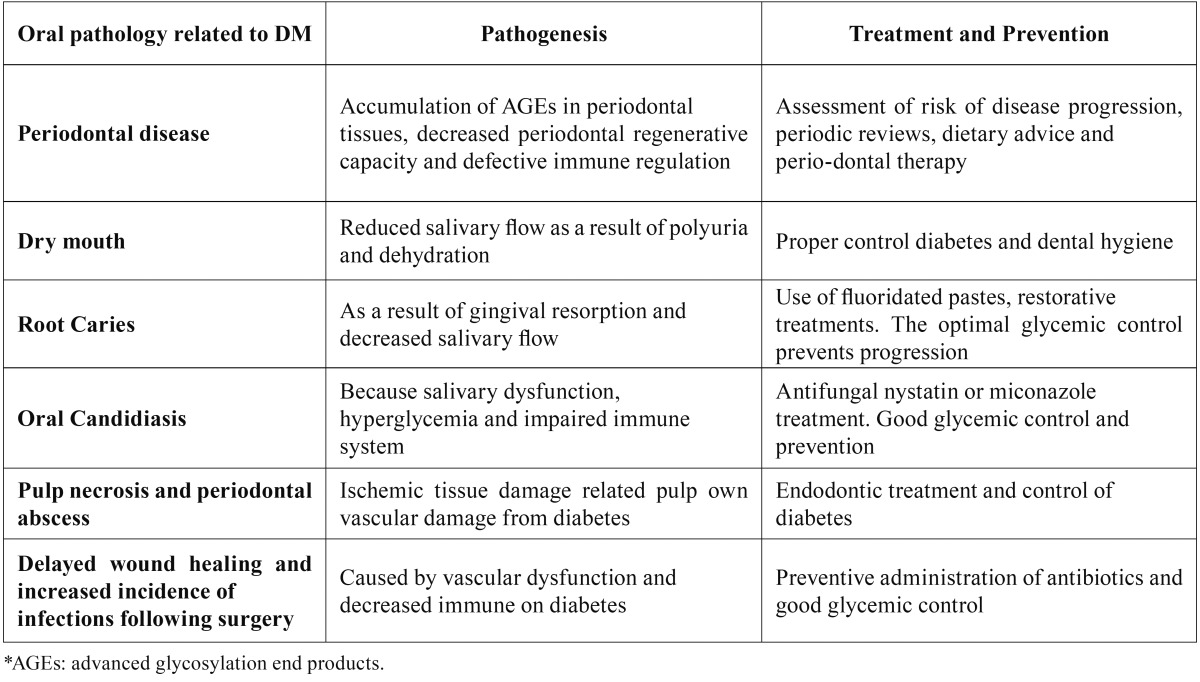
The pathophysiology, treatment and prevention aspects of orofacial diseases related to diabetes (30).

- Oral manifestations of Diabetes Mellitus

For years, research into diabetes has explored the many clinical implications of this highly prevalent disease. As previously mentioned, these include the need for periodontal control as tissue destruction may be accelerated among diabetics, and early management of oral infection will avoid exacerbating the existing metabolic imbalance. It has been found that an individual with uncontrolled diabetes presents a higher risk of infection, as well as abnormal prolonged healing time that will endanger the health of the oral cavity. Research has established that patients with DM may present a variety of oral manifestations ( Table 3). Each of these oral manifestations and their relationship to DM are summarized below:

**Table 3:**
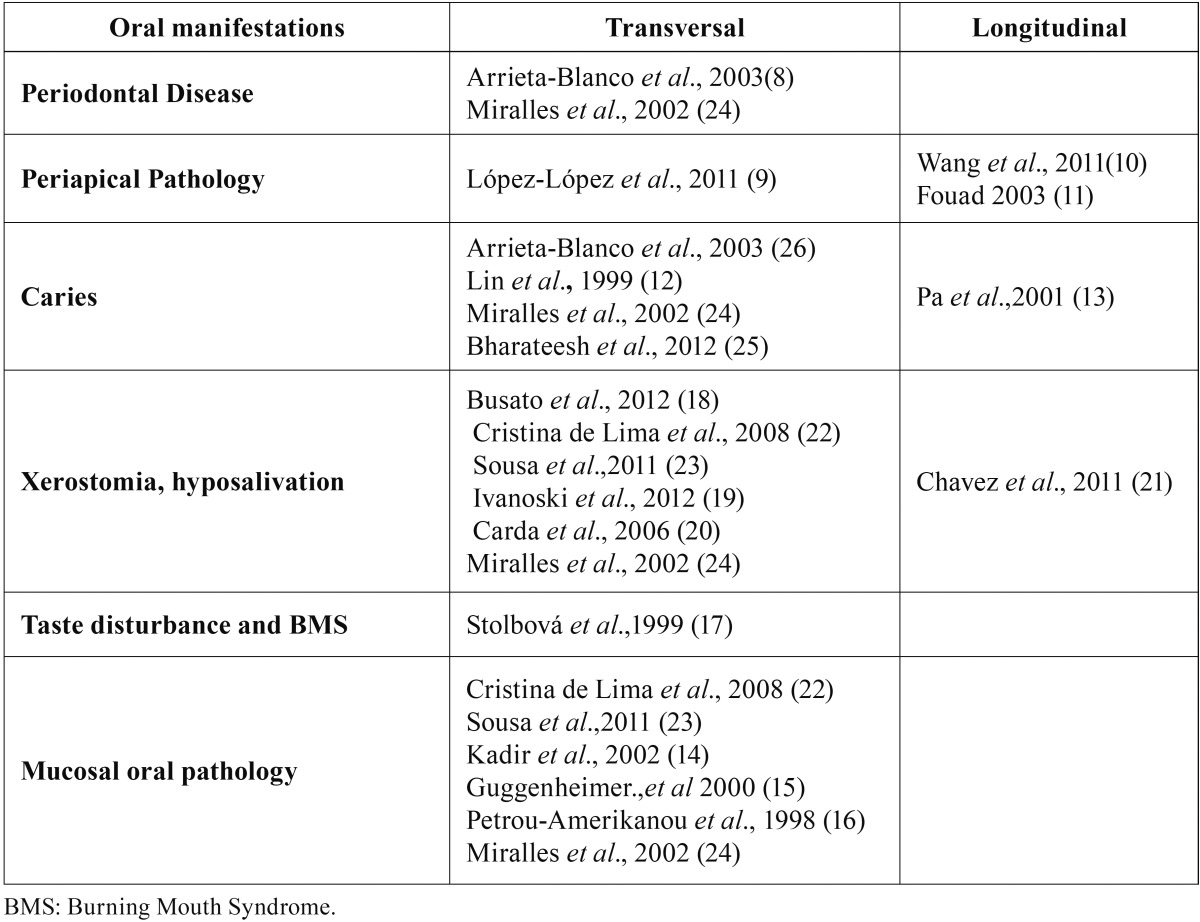
Significant oral manifestations related to diabetes.

i. Diabetes and Periodontal Disease

The main oral complication attributed to diabetes is periodontal disease (PD), considered the sixth complication of DM (31). Simple chewing can cause systemic dissemination of periodontal pathogens and their metabolic products in patients with periodontal disease causing endotoxemia or bacteremia, which results in an increase in serum levels of inflammatory mediators such as Inter-leukin 6 (IL-6), fibrinogen, and C-reactive protein (CRP). Furthermore, systemic inflammation can exacerbate insulin resistance and therefore the management of diabetes. For this reason, correct periodontal treatment can lower the level of proinflammatory mediators, and so contribute to better glycemic control (30).

It has been suggested that there is a degree of synergism between DM and PD. On the one hand, the severity and prevalence of PD increases in diabetics and is worse in diabetics with poor glycemic control. On the other hand, periodontitis may exacerbate diabetes, decreasing glycemic control. However, there is some controversy over this issue; diabetes clearly increases the risk of PD but the impact of PD on glycemic control and the mechanisms by which this occurs are not clear (32).

ii. Diabetes and Periapical Pathology

The scientific literature shows a higher prevalence of periapical lesions in patients with poorly controlled diabetes (28,29). A recent clinical study showed that patients with DM2 presented a significant association with an increased incidence of periapical lesions and endodontic treatments (9). Regarding the success rate of endodontic treatment, an article published in 2011 (33) states that patients with DM had a lower success rate in primary root canal treatment in comparison with non-diabetic patients, while both groups presented the same success rate in secondary root canal treatment (33). Another study found that patients with diabetes are at increased risk of the need for tooth extraction following endodontic treatment. This risk increases in patients with hypertension as well as DM and /or coronary artery disease (10).

The dental pulp of diabetic patients may have limited dental collateral circulation, impaired immune response, and an increased risk of infection or pulp necrosis. Regarding molecular pathology, hyperglycemia is a stimulus for bone resorption, inhibition of osteoblast differentiation, and a reduced capacity for bone recovery (34). It has been observed that the removal of periodontal inflammation can reduce the dose of insulin required for the patient’s glycemic control. For this reason, it is essential to remove all dental pulp infections (28). The special characteristics of periapical lesions in patients with diabetes provide evidence that the treatment objectives and definition of success should be different for these patients. A recent review concluded that current knowledge about the microbiology of endodontic infections and inflammatory reactions is limited, and that such knowledge could help implement new forms of treatment for these patients. Further research is needed to better understand the issue and so increase the success rates of endodontic treatment among these patients (34).

iii. Diabetes and Dental Caries

Information presented in the literature about the relationship between the DM and tooth decay is inconsistent (35). Arrieta-Blanco *et al.* (26) in a study of 144 patients (70 diabetic and 74 non-diabetic) found no significant difference in mean caries between the two groups. The prevalence of carious lesions was 7.39% in diabetic patients and 6.91% in non-diabetics (26). Another study with a sample of 600 patients (300 with diabetes and 300 healthy) showed that the prevalence of dental caries was higher in non-diabetics (32.3%) than in diabetics (13.6 %) (25). As shown in  Table 4, patients with DM had greater need for treatment than healthy subjects, but nevertheless presented a lower rate of tooth decay. Bharateesh *et al.*, suggest that patients with DM may have fewer cavities due to the content of their diet which usually contains more protein and fewer fermentable carbohydrates (25). Another similar study did not find differences in the number of cavities between patients with DM1 and a group of healthy subjects (24).

**Table 4:**
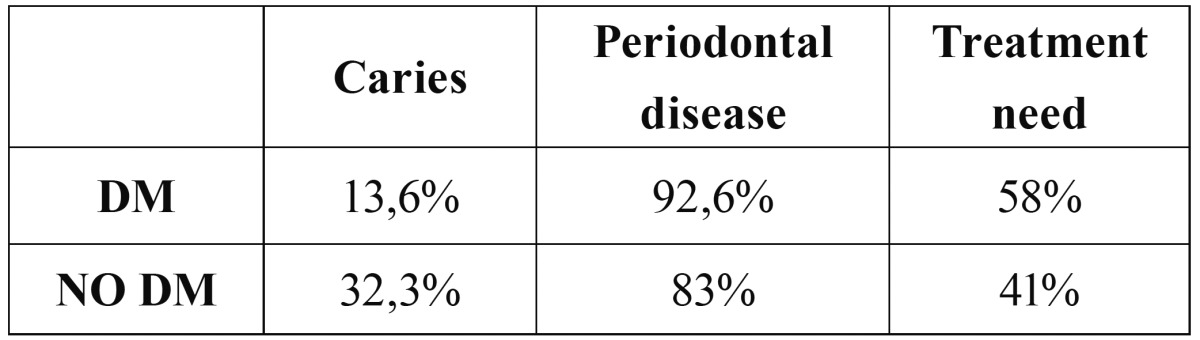
Results study of Bharateesh (25).

Meanwhile, other studies have found a higher incidence of dental caries in patients with DM (12,13), which could be explained by the decrease of salivary secretion suffered by diabetics.

iv. Diabetes and Oral Mucosa

DM Patients may have a higher prevalence of mucosal disorders possibly associated with chronic immunosuppression, delayed healing, and/or salivary hypofunction (14). These alterations include: oral fungal infections such as oral candidiasis (15); fissured tongue, irritation fibroma, traumatic ulcers and lichen planus (16). However, there is some controversy on this issue, as other studies have found no association between DM and candidiasis, or other oral mucosa lesions (22-24).

v. Diabetes and Xerostomia

In a study conducted by Chavez *et al.* (21), a tendency for salivary flow to decrease was observed when HbA1c values increased. A recent study compared the salivary characteristics in 30 patients with diabetes compared with 30 healthy subjects. Eighty per cent of DM patients presented xerostomia, but only 10% of healthy subjects. Furthermore, urea and glucose levels in saliva were significantly higher in diabetics than healthy subjects. This suggests that DM can cause xerostomia and that there may be a significant correlation between the degree of xerostomia and glucose levels in saliva. In addition, increased salivary glucose promotes the proliferation and colonization of bacteria in the oral cavity, and glucose is the basis for Candida development and decreases the activity of neutrophils (19). Another study of 102 patients showed a significant association between DM1 and xerostomia but the results showed that clinical status and salivary conditions did not affect the presence of xerostomia (18). Carda *et al.* (20), in a study of 33 patients, found a significantly higher percentage of xerostomia in patients with DM than in the control group (76.4% and 18.7% respectively). However other studies have not found significant differences in salivary flow between diabetics and non-diabetics (22,23).

vi. Diabetes and Taste disturbance

Taste detection follows a hereditary pattern, but can be influenced by the appearance of neuropathies. When this sensory dysfunction occurs, it can inhibit the ability to maintain a proper diet and can lead to poor glycemic control. Taste alteration has been associated with diabetes and the development of obesity (36). In this context, a 1999 clinical study investigated 73 patients with DM2, 11 patients with DM1, 12 obese patients (BMI> 30) without DM, and 29 control subjects. All subjects underwent electrogustometric examination. The results found hypogeusia in 40% of DM2 patients, in 33% of DM1 patients, 25% of obese patients, while no case of hypogeusia was found in the control group. Ageusia was observed in 5% of DM2 patients, 3% of DM1 patients, and 14% of obese patients. These results suggest that impaired taste may evoke hyperphagia, and then later, obesity (17).

vii. Diabetes and Burning Mouth Syndrome

Burning mouth syndrome (BMS) is characterized by a burning sensation in the oral mucosa and an absence of clinical signs. Its etiology includes systemic, local, and psychological factors (stress, anxiety and depression). It is more common in women, and the average age of the typical SBA patients is 50 to 60 years old (37). Patients with diabetes often have burning mouth syndrome, but a clear relationship between DM and BMS has not been identified (38).

- Knowledge of the relationship between diabetes and oral health

Knowledge and understanding of diabetes and periodontal health is low among diabetic patients, and most are unaware of the oral health complications deriving from the disease they suffer and of the need for proper preventive care. This was reflected in a recent study in which questionnaires were issued to a random sample of 500 diabetic patients. Twenty-eight per cent of patients said they did monitor their periodontal health with regular visits to the dentist; 48% were conscious of the increased susceptibility to gum disease and oral health complications; 38% recognized that periodontal health can affect blood sugar levels (39). Another study of 101 patients with DM1 and DM2, found that 84% of the increased risk of heart disease, 98% of the risk of eye disease, 99% of the risk of circulatory problems and 94% of the risk of kidney disease but only 33% of the participants were aware of the increased risk of periodontal disease. The participants who were aware of the increased risk of periodontal disease had obtained this information from a dentist. The study also found a significant association between metabolic control and dental status (40).

Awareness and understanding of the possible associations between diabetes, oral health and general health need to be increased among diabetic patients. Dentists, doctors and other health professionals should conduct periodontal screening every time a diabetic patient attends a check-up, and should recommend attending regular check-ups by a specialist (39,40). All the evidence registered in the present review highlights the importance of preventive and therapeutic control of DM and periodontal disease. The involvement of oral health professionals in strategies aimed at identifying individuals at risk from diabetes should be max-imized in order to retard the development of possible complications (36).

As for the dentist’s involvement in these strategies, he/she should bear in mind that diabetes is a common disease with concomitant oral manifestations that can modify dental care needs. In this context, dentists must be completely familiar with diagnosis and prevention techniques (39,40). Effective management of diabetic patients requires cooperation between the patient, the doctor, the dentist, and other healthcare professionals. Regular check-ups will allow dentists to anticipate patient needs and interact competently with other healthcare professionals. Careful examination of the oral cavity may discover indications of an underlying systemic condition, and allow early diagnosis and treatment. The examination should include an assessment of changes to the mucosa, periodontal inflammation, and bleeding, as well as the general state of the teeth.

## Conclusions

Diabetes Mellitus leads to multiple complications, which increase when glycemic control of the patient is inadequate. This makes management and prevention important. It has been shown that diabetes exists in a bidirectional relationship with periodontal disease and may lead to other oral pathologies. For this reason, doctors and dentists must be vigilant with regard to the various oral manifestations of diabetes in order to make an early diagnosis.

Full understanding and awareness of the pathophysiology, manifestations, and management of different types of diabetes-related orofacial infection by the endocrinologist and the dentist are essential to optimizing the care of diabetic patients.
